# Water-Drinking and Its Effects

**Published:** 1905-05-06

**Authors:** 


### WATER-DRINKING AND ITS EFFECTS.

Dr. H. Labbé[1] has written to bewail the comparative apathy with which the liquid constituents of diet are regarded by the world at large, especially as regards the quantities necessary for the maintenance of health. He complains, with some justice, that a disproportionate amount of our dietetic interest is lavished upon the solid elements, and asserts his conviction that a healthy adult should never allow his ingestion of liquids to fall below two litres a day. By a somewhat curious coincidence, Dr. P. B. Hawk[2] has published almost synchronously the results of a detailed and careful investigation of the results of copious water-drinking. The purpose of the study was to demonstrate the effects of such a regimen on the excretion by human subjects of nitrogen, sulphur, and phosphorus, as indices of the tissue changes set up by the practice.

The method was briefly as follows. The subjects, healthy young adults, were placed upon a constant diet, and by means of a preliminary period of sufficient length, were brought to an approximate equilibrium as regards the intake and output of nitrogen, sulphur, and phosphorus. This having been achieved, the constant diet was supplemented for two days by a large quantity of water at stated intervals, and a return finally made to the original diet-scale. The constant diet was made up as follows: Butter, 75 grammes; crackers, 330 grammes; whole milk, 1,800 grammes; water 500 cc. On the two days of excessive water-administration an additional quantity amounting to 4,500 cc. of this liquid was given on each day. Throughout the experiment a careful daily estimate was made of the excretion of nitrogen, sulphur and phosphorus by the urine and fæces. This estimate revealed the following facts.

In all cases the first day of excessive water-ingestion resulted in a great increase in the excretion of urea—an increase to the extent of 12 per cent. as compared with the preliminary totals, which increase fell to 6 per cent. on the second day.

The excretion of sulphur varied very similarly to that of urea, but the phosphorus excretion showed a remarkable difference. It was, like the others, increased in quantity, but instead of reaching its maximum on the first day of the excessive water supply, reached it in every instance on the second.

Results similar to these have been recorded by various observers, and a question arises as to the interpretation of the facts. Are we to consider that the undue amount of liquid has merely flushed from the tissues a quantity of waste products which are untouched by a normal concentration of the blood, or are we to suppose that the action of the superfluous liquid has increased the tissue metabolism? The author, in view of the constancy with which the nitrogen and sulphur excretion reaches a maximum on the first day, inclines to the opinion that the increase, as far as they are concerned, depends primarily upon a mechanical removal of pre-existing waste from the tissues, and in a minor degree to some slight increase in proteid katabolism; the increase in phosphorus excretion, on the other hand, he regards as evidence that copious water-drinking enhances the activity of the metabolism of nucleins, lecithins, and other phosphorus-containing cellular elements. Finally, he sees nothing in the experiments to lead to the supposition that large volumes of water are in any way detrimental, but on the contrary inclines to advocate their ingestion. He states that such large quantities increase peristalsis, promote the biliary secretion, increase the arterial tension, and reduce the body heat. The increase of arterial tension suggests itself as a possible source of danger in certain pathological conditions, but it seems fairly clear that a healthy man can hardly over-drink himself on water.

[1] La Presse Médicale, April 15, 1905. [2] Univ. of Pennsylvania Med. Bulletin, March 1905.

